# Leprosy post-exposure prophylaxis in the Indian health system: A cost-effectiveness analysis

**DOI:** 10.1371/journal.pntd.0008521

**Published:** 2020-08-04

**Authors:** Anuj Tiwari, David J. Blok, Mohammad Arif, Jan Hendrik Richardus

**Affiliations:** 1 Department of Public Health, Erasmus MC, University Medical Center Rotterdam, Rotterdam, The Netherlands; 2 NLR India, New Delhi, India; Adolfo Lutz Institute of Sao Jose do Rio Preto, BRAZIL

## Abstract

India has the highest burden of leprosy in the world. Following a recent WHO guideline, the Indian National Leprosy Programme is introducing post-exposure prophylaxis with single-dose rifampicin (SDR-PEP) in all high-endemic districts of the country. The aim of this study is to estimate the long-term cost-effectiveness of SDR-PEP in different leprosy disability burden situations. We used a stochastic individual-based model (SIMCOLEP) to simulate the leprosy new case detection rate trend and the impact of implementing contact screening and SDR-PEP from 2016 to 2040 (25 years) in the Union Territory of Dadra Nagar Haveli (DNH) in India. Effects of the intervention were expressed as disability adjusted life years (DALY) averted under three assumption of disability prevention: 1) all grade 1 disability (G1D) cases prevented; 2) G1D cases prevented in PB cases only; 3) no disability prevented. Costs were US$ 2.9 per contact. Costs and effects were discounted at 3%.

The incremental cost per DALY averted by SDR-PEP was US$ 210, US$ 447, and US$ 5,673 in the 25^th^ year under assumption 1, 2, and 3, respectively. If prevention of G1D was assumed, the probability of cost-effectiveness was 1.0 at the threshold of US$ 2,000, which is equivalent to the GDP per capita of India. The probability of cost-effectiveness was 0.6, if no disability prevention was assumed. The cost per new leprosy case averted was US$ 2,873. Contact listing, screening and the provision of SDR-PEP is a cost-effective strategy in leprosy control in both the short (5 years) and long term (25 years). The cost-effectiveness depends on the extent to which disability can be prevented. As the intervention becomes increasingly cost-effective in the long term, we recommend a long-term commitment for its implementation.

## Author summary

Leprosy is an infectious disease caused by the *Mycobacterium leprae* and is one of the major causes of preventable disability in low- and middle-income countries. Disabled people face lifelong socioeconomic challenges and are often excluded from society. It brings people affected by leprosy in a vicious cycle of poverty and disability. This cycle needs to be curbed through scientific evidence on health economic aspects of leprosy. Unfortunately, there is not much literature available on cost or cost-effectiveness of leprosy prevention. New prevention methods such as post-exposure chemoprophylaxis are now available, providing opportunity to collect up-to-date health economic evidence to support health policy and planning. Here, we present a cost-effectiveness study of contact screening in combination of post-exposure prophylaxis with single-dose rifampicin in the Union Territory of Dadra Nagar Haveli in India. We compare the intervention with the continuation of the routine leprosy control programme. As leprosy is moving towards elimination, evidence from this study will contribute to an investment case for leprosy elimination.

## Introduction

Leprosy is an infectious disease caused by *Mycobacterium leprae* affecting mainly the skin and peripheral nerves, and may lead to life-long disability when untreated. Three disability grades are recognized: grade 0 (no disability); grade I disability (G1D); and grade II disability (G2D), the latter being more severe including visible deformities. Globally, 208,619 new cases were detected in 2018 [1]. Due to a long incubation period [2], an infected person may remain asymptomatic and undetected for a long time and can transmit the bacteria to others. The introduction of multidrug therapy (MDT) in the 1980s substantially decreased prevalence of the disease, but the new case detection rate (incidence) remained almost stagnant [3]. Therefore, the goal of leprosy elimination and past investments into this goal are at risk [4].

In India, 128/682 (18.7%) districts are still reporting more than one new case per 10,000 population. The National Leprosy Eradication Programme (NLEP) aims to eliminate leprosy in these high-endemic districts [5]. There are, however, important challenges to interrupt transmission of *M*. *leprae*. The current leprosy burden is underestimated and needs correction by accounting for hidden cases [6, 7]. Active case finding needs to be intensified for early detection and treatment. The available prevention methods, such as post-exposure prophylaxis through single-dose rifampicin (SDR-PEP) needs a scale-up to demonstrate its impact. This is challenging [8], as countries need information for the introduction of new interventions such as on the cost-effectiveness, duration of implementation, expected outcomes, and uncertainties.

SDR-PEP was field-tested in the Union Territory of Dadra and Nagar Haveli (DNH) between 2015 and 2018 as part of the Leprosy Post-Exposure Prophylaxis (LPEP) program [9]. The LPEP program was designed to assess the feasibility and impact of contact tracing and SDR-PEP to asymptomatic contacts of leprosy cases [10]. The necessary complementary activities to the routine leprosy programme were contact listing, screening, and follow-up. Additionally, LPEP also increased the awareness of leprosy in the community [9]. In DNH a total of 30,295 contacts received SDR-PEP between 2015 and 2018. The distribution of SDR-PEP is currently ongoing, but now as a routine activity under NLEP.

The LPEP program systematically captured relevant data that can guide the scale-up of the intervention [9, 11]. The actual impact of SDR-PEP however, is difficult to observe in a three-year programme, because of the existing backlog of leprosy cases. Therefore, we use mathematical modelling to estimate the long term impact of SDR-PEP on the NCDR. The aim of this study is to estimate the long-term cost-effectiveness of SDR-PEP in different leprosy disability burden situations. The results can assist governmental and non-government organisations in planning their investment for leprosy control and ultimately contribute to a global investment case for leprosy elimination [12].

## Methodology

### Ethics statement

The study was conducted under the Leprosy Post Exposure Prophylaxis (LPEP) program, approved in India by the Institutional Human Ethics Committees of the National Institute of Epidemiology (NIE/IHEC201407-01).

### Study setting

DNH is highly endemic for leprosy with the highest annual new case detection rate (ANCDR) in India in 2017 [13, 14]. Until recently DNH also had a high number of child cases, which is considered an indication of active transmission of *M*. *leprae* [13]. More than half of the DNH population is tribal (Census 2011), living mostly in rural areas with limited resources. Despite the highest ANCDR in DNH, the G2D rate is low (0.37% of all new cases) compared to other endemic parts of India [14]. In DNH, the uptake of leprosy services and public health expenditure is better than the neighbouring areas [15, 16]. S1 Table provides detailed information on demography, socioeconomics and epidemiology of DNH.

### Model description

We used the stochastic individual-based model SIMCOLEP to predict trends of the new cases per 100,000. The model simulates life-histories of individuals and the spread of *M*. *leprae* in the population in DNH. Transmission can occur when an infectious individual has contact with a susceptible individual. Two transmission processes are modelled separately: transmission in the general population and within-household. The latter reflects the increased risk of transmission among close contacts. The natural history of leprosy was modelled following the same methodology as described in Fischer *et al*. 2010 and Blok *et al*. 2015 [17, 18]. In the model, infected individuals can develop either paucibacillary (PB) or multibacillary (MB) leprosy following the observed MB / PB ratio (i.e. 26/74). In the model, PB leprosy cases are assumed to self-heal with a rate of 20% per year, while MB leprosy remains chronic until detection and treatment (S2 Table). Leprosy control includes passive case detection and treatment with multidrug therapy (MDT), and additional active case finding activities such as contact tracing and survey. In the model, the quality of the control programme is reflected by detection delays, and active case finding activities are specified using annual coverage rates.

The model was fitted to the leprosy trends in DNH. The model was quantified with demographic data from the census (S3 Table). The model was then fitted to the household size distribution in DNH (S1 Fig). The transmission risk (i.e. contact rate) and the passive case detection delays mimic historic NCDR trends (S2 Table). Data were used from NLEP for the period 1995–2015. The model was first calibrated to data points until 2012, then validated for the years 2013–2015 (S2 Fig). After validation, the model was calibrated using the complete dataset. This was regarded as the situation of continuation of the routine national programme prior to the introduction of the LPEP program. Projections of the NCDR trend were based on 1,000 runs.

### Modelled scenarios

We compared two scenarios: A) the SDR-PEP intervention; and B) the continuation of the routine programme. The SDR-PEP intervention represents the LPEP program in addition to the ongoing NLEP in DNH. The necessary complementary activities to the routine leprosy programme were contact tracing, screening, and follow-up. SDR-PEP was provided to contacts without leprosy or other contra-indications. A contact detected with leprosy during screening was referred for MDT treatment. Contacts were listed retro- and prospectively. Retrospective tracing includes contacts from leprosy patients diagnosed up to 2 years prior to the intervention programme in 2015. Additionally, the programme also increased the awareness of leprosy in the community [9]. On average, 26 contacts were screened per index patient. Based on results from the COLEP trial, we assumed that the effectiveness of SDR-PEP was higher among neighbours and social contacts (70%) than household contacts (50%) [19].

Predictions of the number of new MB and PB leprosy cases were made from 2015 until 2040 for the SDR-PEP intervention and the routine programme scenario (S3 Fig). Using age proportions of leprosy patients in DNH of the last 6 years (2013–2018), the modelled new MB and PB leprosy cases were apportioned into five age groups: 0–4, 5–14, 15–44, 45–59, and 60+ years. We also predicted the annual number of contacts that received SDR until 2040 (S4 Fig).

### Disability-adjusted life years

Health effects were measured as disability adjusted life years (DALYs). Leprosy disability weights were obtained from the global burden of disease study (GBD 2017), which were 0.011 for G1D and 0.067 for G2D [20]. We assumed that disability is irreversible and that leprosy does not cause any mortality; therefore, the DALY is equal to years lived with disability (YLD). The DALYs were calculated as follows:

$$DALY(t)=\sum_{a=1}^{n_{a}}\left(I_{G1D}(a,t)∙D_{G1D}∙(a)\right)+(I_{G2D}(a,t)∙D_{G2D}∙L(a))$$

With:

*n_a_* = number of age groups (0–4, 5–14, 15–44, 45–59, 60+ years);

*I*_*G*1*D*_(*a,t*)/*I*_*G*2*D*_(*a,t*) = Number of cases with G1D / G2D per 100,000 in age group *a* at time *t*;

*D*_*G*1*D*_/*D*_*G*2*D*_ = Disability weights for G1D/G2D;

*L*(*a*) = Life Expectancy of age group *a*. Data obtained from SRS Based Life Table 2011–15, Census of India

The annual number of new cases with G1D and G2D in the SDR-PEP intervention and routine scenario were estimated using the modelled new MB and PB leprosy cases per year. Since no data were available on the proportion of new cases with G1D and G2D among PB and MB leprosy cases, we made the following assumptions to calculate the number of cases with G1D and G2D:

1. 3.6% of total leprosy cases have G2D (following the reported statistics on leprosy in India [14]; all G2D cases emerge from MB leprosy cases.
2. All remaining MB leprosy cases have G1D.
3. 50% of the total PB leprosy cases have G1D.
4. All remaining PB leprosy cases have no disability.

As the SDR-PEP intervention scenario includes active contact tracing and screening, it is highly likely that the time until diagnosis would be reduced, which would prevent (progression to) disability. To account for this in the SDR-PEP intervention, we calculated DALYs under three assumptions of disability prevention (Table 1):

1. Prevention of all G1D cases.
2. Prevention of G1D in PB cases only.
3. No additional prevention (i.e. same as routine scenario).

**Table 1:** Assumptions to estimate disease burden in the routine and SDR-PEP intervention scenario.

Assumptions	Routine	SDR-PEP intervention		
		Assumption 1: All G1D cases prevented	Assumption 2: G1D cases prevented in PB cases only	Assumption 3: No disability prevented
1. 3.6% of total leprosy cases have G2D (emerged from MB leprosy cases)	Included	Included	Included	Included
2. All remaining MB leprosy cases have G1D	Included	Prevented by intervention	Included	Included
3. 50% of PB leprosy cases have G1D	Included	Prevented by intervention	Prevented by intervention	Included
4. All remaining PB leprosy cases have no disability	Included	Included	Included	Included

Note: The shaded cells indicate whether the assumption was included in the analysis or not

### Costs

Costs of the intervention included the cost of contact listing, tracing, screening, and drug, and were calculated from the perspective of the health system. The composite cost for SDR-PEP intervention was estimated to be US$ 2.9 (95% CI: 2.5–3.7) per contact [16]. The cost per contact was sampled 1,000 times and multiplied with the modelled number of contacts that received SDR-PEP to calculate the total costs of the intervention in DNH per year.

### Cost-effectiveness analysis

Cost-effectiveness of the SDR-PEP intervention was assessed using incremental cost-effectiveness ratios (ICERs):

$$ICER=\frac{{Cost}_{SDR-PEP}-{Cost}_{Routine}}{{DALY}_{SDR-PEP}-{DALY}_{Routine}}$$

DALYs and costs were cumulative and both discounted at 3%. The time horizon was 25 years, i.e. 2016 to 2040, and cost-effectiveness was assessed at five-year intervals. We applied a willingness to pay (WTP) threshold equal to one and three times the GDP per capita of India 2017 as, i.e., $2,000 and $6,000, respectively, following the suggestion of the WHO [21]. If results were cost-effective at $2,000, we did not present the results using a threshold of $6,000. Using cost-effectiveness acceptability curves (CEAC), we assessed the probability of the SDR-PEP intervention to be cost-effective against a range of willingness-to-pay thresholds. This depicts the uncertainty associated with the results. [21, 22]. Additionally, we also calculated the incremental cost per new leprosy case averted by SDR-PEP. We used the BCEA package in the software R 3.6.1 for the analysis [23].

## Results

**Fig 1:**
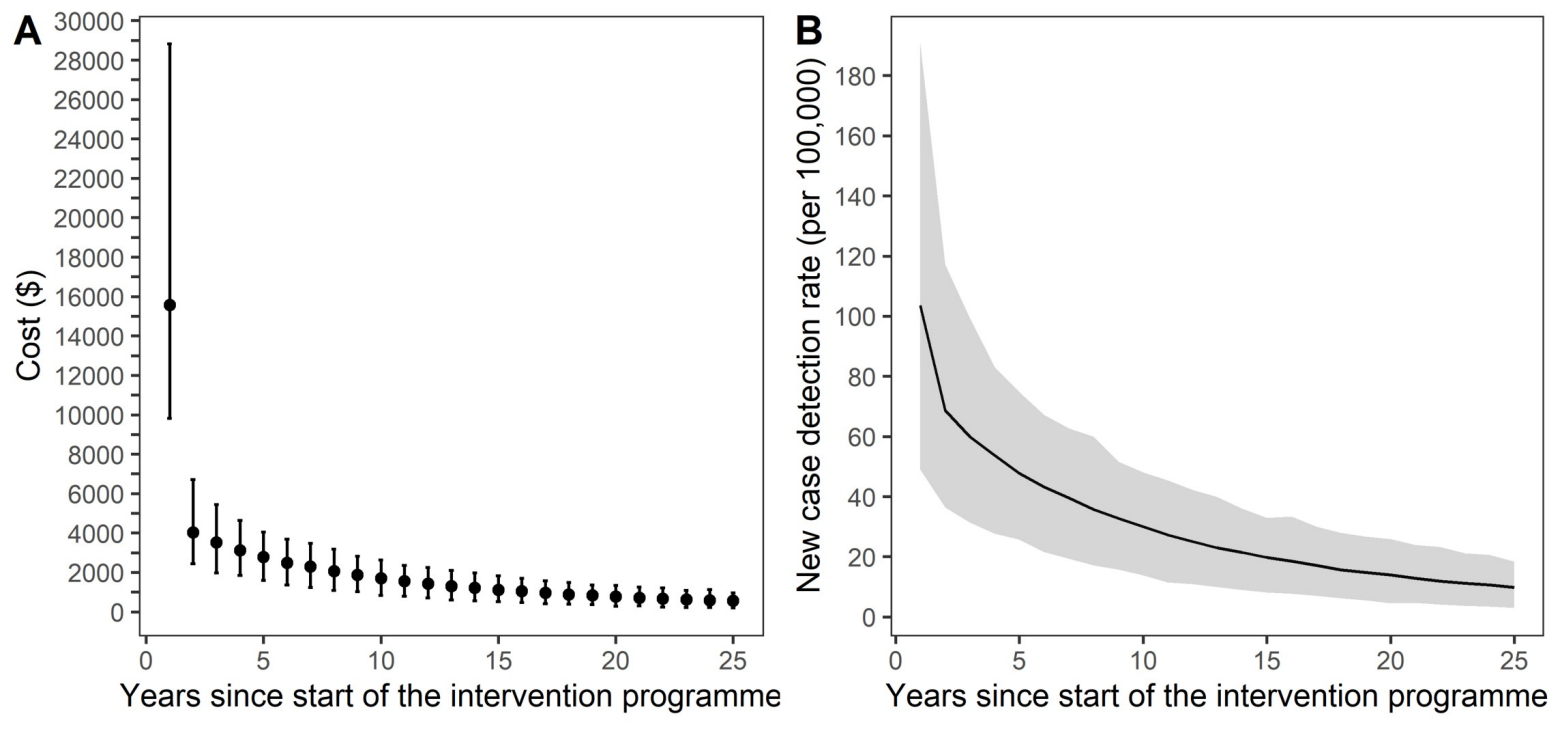
**SDR-PEP intervention (A) estimated cost and (B) predicted new cases detection rate per 100,000 persons.** (A) *Points represent the mean cost in US$ per year and the error bars represent the 95% uncertainty interval based on 1*,*000 simulation runs*. *(B) The black line represents the mean new case detection rate and shaded region represents the 95% uncertainty interval based on 1*,*000 simulation runs*.

Fig 1 shows the trend in annual cost of SDR-PEP implementation for the next 25 years (Fig 1A) and model predicted new cases detection rate per 100,000 persons (Fig 1B). As the number of SDR-PEP for contacts depends on the number of new cases, both graphs showed a declining trend. In the first year, the cost was on average $16,000, but subsequently dropped to around $600 in the last five years due to the decreasing numbers of new leprosy patients. On average, the cost decreased by 11% annually over 25 years. The percentage decrease was highest in the 2^nd^ year (74.1%) and least in the 23^rd^ year (4.9%).

**Fig 2:**
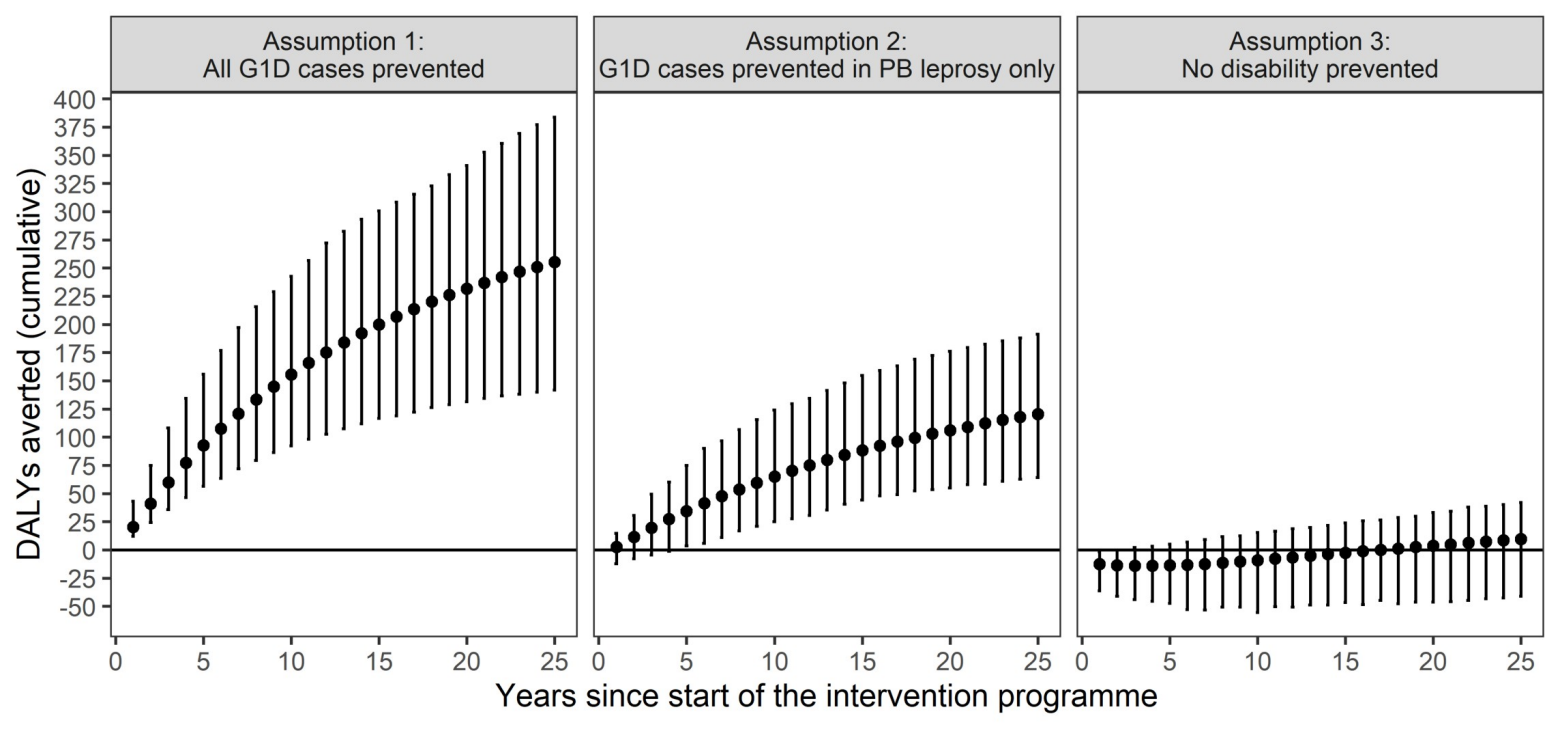
Estimated cumulative DALYs averted as a result of the SDR-PEP intervention under three assumptions of disability prevention. Points represent the mean cumulative DALYs averted per year. The error bars represent the 95% uncertainty interval based on 1,000 simulation runs.

Fig 2 shows the trend in averted DALYs as a result of the SDR-PEP intervention under three assumptions of disability prevention. Over a period of 25 years, the number of averted DALYs were 250, 65 and 10 when assuming prevention of all G1D cases, prevention of G1D case in PB leprosy cases and no prevention of disability, respectively.

**Fig 3:**
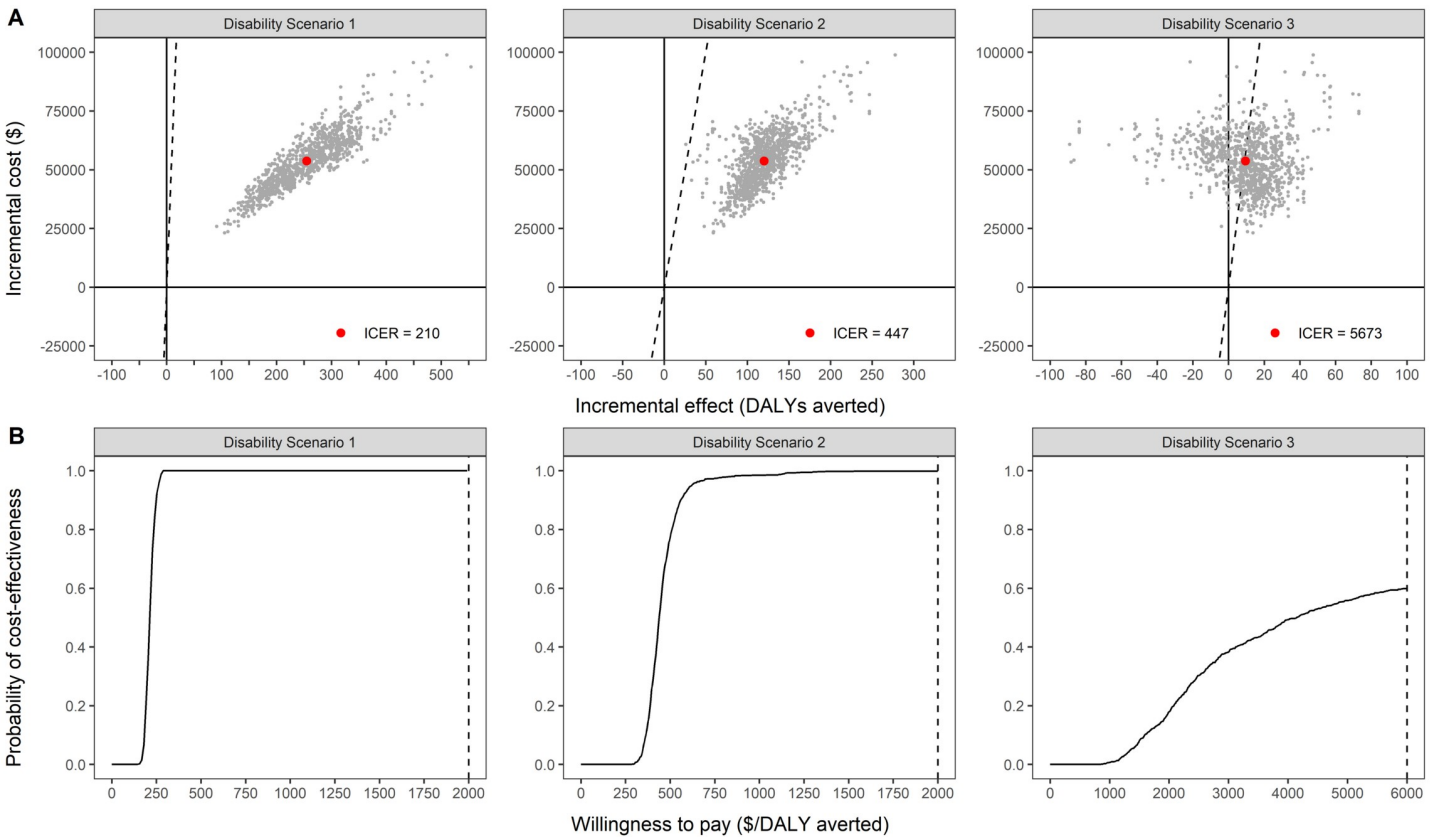
Cost-effectiveness plane and acceptability curve of assumption 1–3 at 25^th^ year. (A) Red dot represents the incremental cost-effectiveness ratio, and dotted line represents the threshold. (B) Blackline represent the cost-effectiveness acceptability curve.

Fig 3A presents the incremental cost and effect in the 25^th^ year of the intervention. The incremental cost to avert one DALY was US$210, US$447 and US$5,673 if we assumed prevention of all G1D cases, prevention of G1D case in PB leprosy cases and no prevention of disability, respectively. The probability of cost-effectiveness at a willingness to pay threshold of US$2,000 was 100% in the two scenarios with assumed prevention of disability (Fig 3B). In the scenario without prevention of disability, there is 60% chance that the intervention is cost-effective given a willingness-to-pay threshold of US$ 6,000. The cost per new leprosy case averted was US$ 2,873.

Table 2 provides results at five-year time intervals. In the assumption 1 and 2, the incremental cost to avert a DALY remained under the WTP threshold of US$ 2,000 in all the intervals. There was a high probability (CEAC 0.94–1.00) that the result remains cost-effective under uncertainty. In both assumptions, the SDR-PEP intervention becomes more cost-effective with increasing time horizon. In assumption 3, which was conservative in assuming the reduction in disability as a result of the intervention porgramme, the incremental cost-effectiveness ratio crossed US$ 2000 but remained below WTP threshold of US$ 6000. The probability of the intervention to be cost-effective was 0.6 at the 25^th^ year.

**Table 2:** SDR-PEP incremental cost-effectiveness ratio and probability of cost-effectiveness at five year intervals.

Time horizon in years		Assumption 1: All G1D cases prevented*	Assumption 2: G1D cases prevented in PB cases only*	Assumption 3: No disability prevented**
25	ICER (US$/DALY averted)	210	447	5,673
	Probability of cost-effectiveness	1.00	1.00	0.60
20	ICER (US$/DALY averted)	218	476	Dominated
	Probability of cost-effectiveness	1.00	1.00	
15	ICER (US$/DALY averted)	230	521	
	Probability of cost-effectiveness	1.00	0.99	
10	ICER (US$/DALY averted)	253	607	
	Probability of cost-effectiveness	1.00	0.98	
5	ICER (US$/DALY averted)	312	843	
	Probability of cost-effectiveness	1.00	0.94	

* Willingness to pay threshold: $2000

** Willingness to pay threshold: $6000

ICER: Incremental cost-effectiveness ratio

In a sensitivity analysis, we explored what the proportion of G1D prevented should be in order for the intervention to be cost-effective with a 90% probability. The break point is at 40% of G1D prevented in PB cases.

In a sensitivity analysis, we explored what the proportion of G1D prevented should be in order for the intervention to be cost-effective with a 90% probability. The break point is at 40% of G1D prevented in PB cases.

## Discussion

The aim of this study was to estimate the cost-effectiveness of SDR-PEP in different leprosy disability burden situations. The SDR-PEP intervention was very cost-effective when we assumed that G1D cases could be completely or partially prevented. In those cases, the cost-effectiveness was attained within the first 5 years, which indicates that SDR-PEP can be quick in returning the investment. Even when no prevention of disability was assumed, the ICER remained below the willingness-to-pay threshold, but only in the long term.

The annual cost of SDR-PEP implementation was highest at the start of the intervention and decreased sharply over time. The SDR-PEP intervention will not be resource-intensive for a long time and the cost may come down to an affordable level for the general health care system within 7–10 years of implementation (Fig 1A). The ‘cost per new leprosy case averted’ was high, but this measure cannot be considered similar to cost-effectiveness, because WHO guidelines on cost-effectiveness analysis allow conclusions only on ‘DALYs averted’ and not on ‘cases prevented’ [21]. However, the cost per case is useful for programme planning and case base reimbursement.

Our results highlight that the assumptions regarding prevention of disability due to SDR-PEP implementation have a large impact on the cost-effectiveness of the intervention. From literature, we know that active case finding with robust screening reduces detection delay of leprosy, thus preventing disabilities [11]. Additionally, prevention projects increase awareness in communities, which increase self-reporting and further reduce detection delay and disability [24]. The quality of screening is also better when accompanied with the distribution of a drug (SDR-PEP), because of the strict need to establish contra-indications and avoid adverse events [9, 25]. To be conservative, we only included assumptions on preventing G1D in the analysis. Prevention of all G1D can be regarded as optimistic, while prevention of some (e.g. 50%) G1D is a more realistic assumption. We did not assume any prevention of G2D (i.e. 3.6% of total cases remained G2D). It is, however, reasonable to assume that this proportion might go down too, especially in the long term. This would result in a higher number of DALYs averted, especially in areas reporting a high proportion of G2D such as Chhattisgarh (4.3%) and Andhra Pradesh (4.8%) [14]. Even when we assumed no prevention of disability, the ICER was below the WTP threshold of $6,000, which was cost-effective, but with a low probability of cost-effectiveness under uncertainty. However, the gap to achieve a high level of probability was not large.

Besides epidemiology, methodological factors can also influence cost-effectiveness. The first factor is the selection of a threshold. According to the WHO CHOICE guidelines, an ICER below GDP per capita is very cost-effective and below the three times of GDP per capita it is cost-effective [21]. Despite being commonly used, this rule is also criticized [26, 27]. Especially in low and middle income country settings, the suggested WTP threshold might be too optimistic. However, even if we would have assumed a WTP threshold that is equal to 50% of the GDP (i.e. US$ 1,000), the SDR-PEP intervention remains cost-effective if prevention of disability is taken into account. Formally the recommendation is to use the value of a statistical life (VSL). Because VSL accounts for productivity and consumption, it is higher than the GDP per capita [28, 29]. Further, some studies used GDP per capita at purchasing power parity (PPP), which is also higher than the GDP per capita constant [30].

The second factor is the disability weights (i.e. G1D = 0.011 and G2D = 0.067). In fact, these weights are not specific for leprosy and are generally considered to be too low [20]. As a result, the number of DALYs averted in this study is underestimated. It is important that leprosy disability weights should be updated and account more realistically for mental health problems, discomfort and reduced mobility due to leprosy [4, 31]. Nevertheless, our estimates are in-line with GBD 2017 because we used the same disability weights.

To our knowledge, this is the first leprosy specific publication that presents the results of an cost-effectiveness analysis in DALYs averted. Existing leprosy cost-effectiveness studies measured effects in terms of cases or consequences prevented [32–34]. The COLEP study looked at the cost-effectiveness of SDR-PEP in Bangladesh. This study reported an ICER of $158 per leprosy case prevented, ICER $214 in neighbours of neighbours and social contacts, ICER $497 in next-door neighbours, and ICER $856 among household contacts.

There are limitations to this study. First, our analysis has a health system and not a societal perspective. The patient’s opportunity cost to avail the SDR-PEP was not considered in the analysis. Second, the cost-saving by preventing cases and disabilities were not considered in the analysis. Third, data on the G1D proportion out of total disabilities was not available at any level. We could therefore not model this figure, but needed to assume the value. Finally, our results are only applicable to the Indian setting where contact tracing is formally part of the national programme, but its implementation level may vary in different states. The results are not necessarily applicable to other countries as the leprosy epidemiology and cost may vary considerably. We therefore recommend similar studies in the other endemic countries for global generalizability.

### Conclusion

We conclude that contact listing, screening and the provision of SDR-PEP is a cost-effective strategy in leprosy control in both the short (5 years) and long term (25 years). The cost-effectiveness of the SDR-PEP intervention depends on the extent to which disability can be prevented. As the intervention becomes increasingly cost-effective in the long term, we recommend a long-term commitment for the implementation of this intervention.

## Supporting information

S1 Fig The observed and modelled household size distribution. (DOCX)

S2 Fig Model calibration and validation of leprosy epidemiology in Dadra Nagar & Haveli India. (DOCX)

S3 Fig Predicted impact of SDR-PEP intervention in Dadra Nagar & Haveli India S3 Table. Demographic data and parameters to quantify the model.
(DOCX)

S4 Fig Contacts received SDR-PEP. (DOCX)

S1 Table Pre and post LPEP comparison of Dadra and Nagar Haveli (DNH) on demography, socioeconomics and epidemiology. (DOCX)

S2 Table Epidemiologic data and parameters to quantify the model. (DOCX)

S3 Table Demographic data and parameters to quantify the model. (DOCX)
