# Supplementary material for: Leprosy post-exposure prophylaxis in the Indian health system: A cost-effectiveness analysis
Source: PLoS Negl Trop Dis. 2020 Aug 4;14(8):e0008521. doi: 10.1371/journal.pntd.0008521 (PMC7428216; doi:10.1371/journal.pntd.0008521)
Supplement: S1 Fig — (DOCX) [file pntd.0008521.s001.docx]

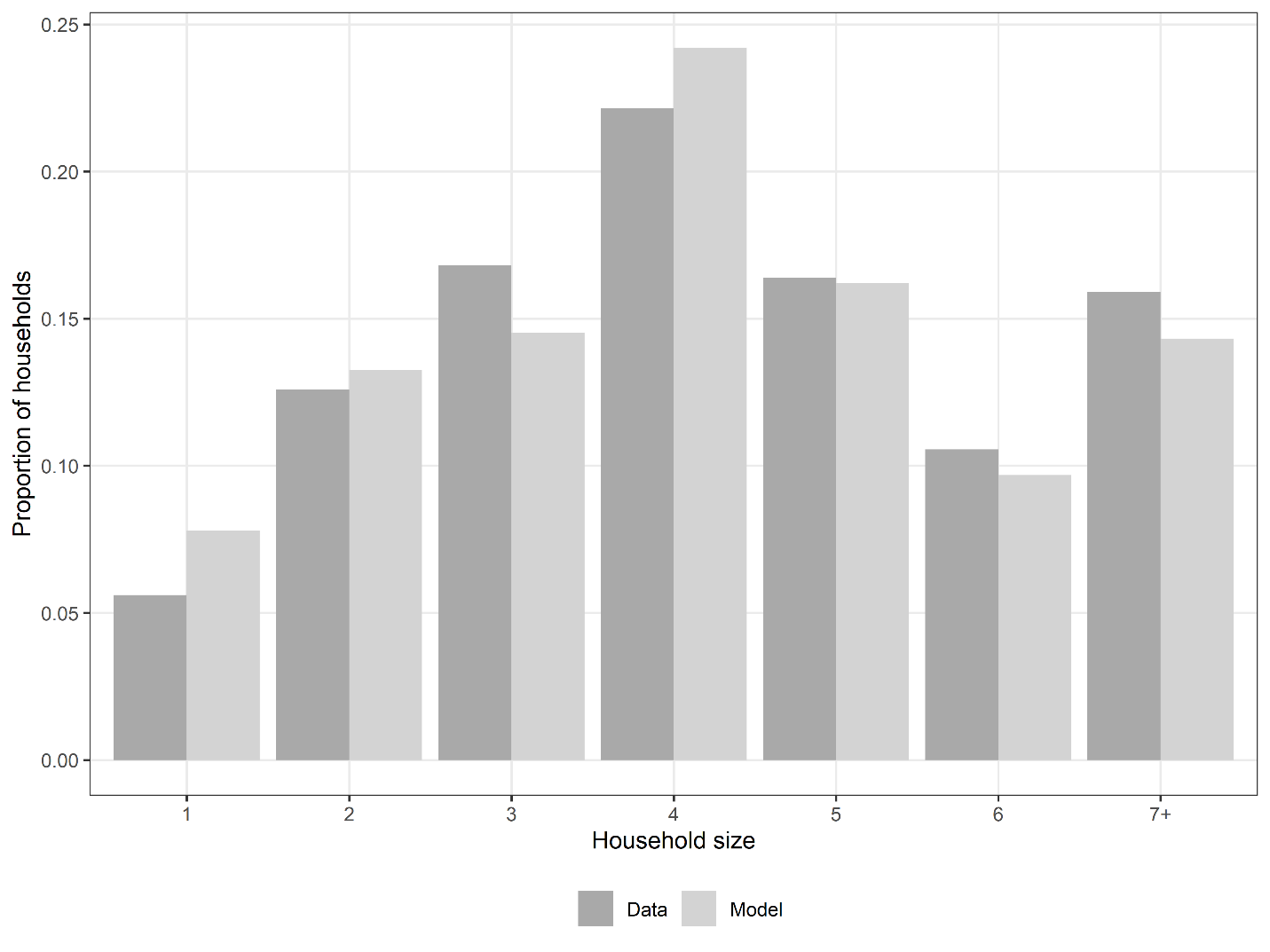


**S1 Fig. The observed and modelled household size distribution.**

The observed distribution is household size distribution of Dadra Nagar Haveli in India in 2011. Data were obtained from Census India (2011). The simulated distribution was obtained by fitting the model to this data. There is no significant difference between data and modeled distribution: India (p = 0.96, χ^2^- test).
