# Supplementary material for: Leprosy post-exposure prophylaxis in the Indian health system: A cost-effectiveness analysis
Source: PLoS Negl Trop Dis. 2020 Aug 4;14(8):e0008521. doi: 10.1371/journal.pntd.0008521 (PMC7428216; doi:10.1371/journal.pntd.0008521)
Supplement: S2 Fig — (DOCX) [file pntd.0008521.s002.docx]

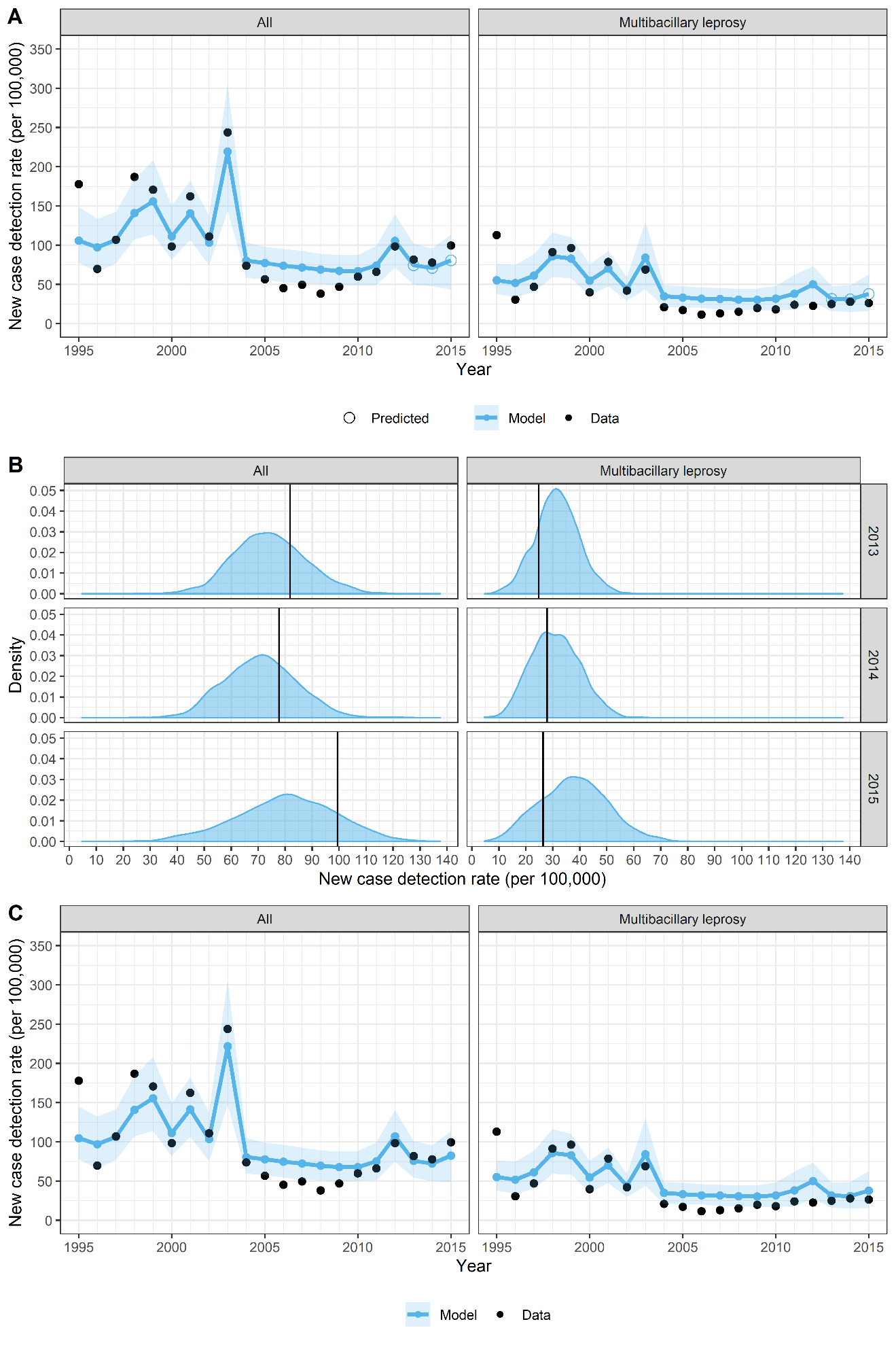


**S2 Fig. Model calibration and validation of leprosy epidemiology in Dadra Nagar & Haveli India**

(A) Comparison of predicted trends with the observed numbers of all new cases and new multibacillary leprosy cases. The model was fitted to the observed cases from 1995 to 2012. Short-term predictions (2013-2015) were evaluated to validate the model. Results are the average of 1000 runs. The shaded area is the 95% uncertainty interval, representing the uncertainty in parameter estimates.

(B) Distribution of predicted numbers of new cases of leprosy in 2013-2015. The observed value for each year is indicated by a vertical black line. The observed data falls within the distribution for each year.

(C) Comparison of predicted trends with the observed numbers of all new cases and new multibacillary leprosy cases. After evaluation, the model was fitted to the complete dataset (1995-2015), which will be used to make predictions beyond 2015. Results are the average of 1000 runs. The shaded area is the 95% uncertainty interval, representing the uncertainty in parameter estimates.
