# Supplementary material for: Leprosy post-exposure prophylaxis in the Indian health system: A cost-effectiveness analysis
Source: PLoS Negl Trop Dis. 2020 Aug 4;14(8):e0008521. doi: 10.1371/journal.pntd.0008521 (PMC7428216; doi:10.1371/journal.pntd.0008521)
Supplement: S3 Fig — Demographic data and parameters to quantify the model. (DOCX) [file pntd.0008521.s003.docx]

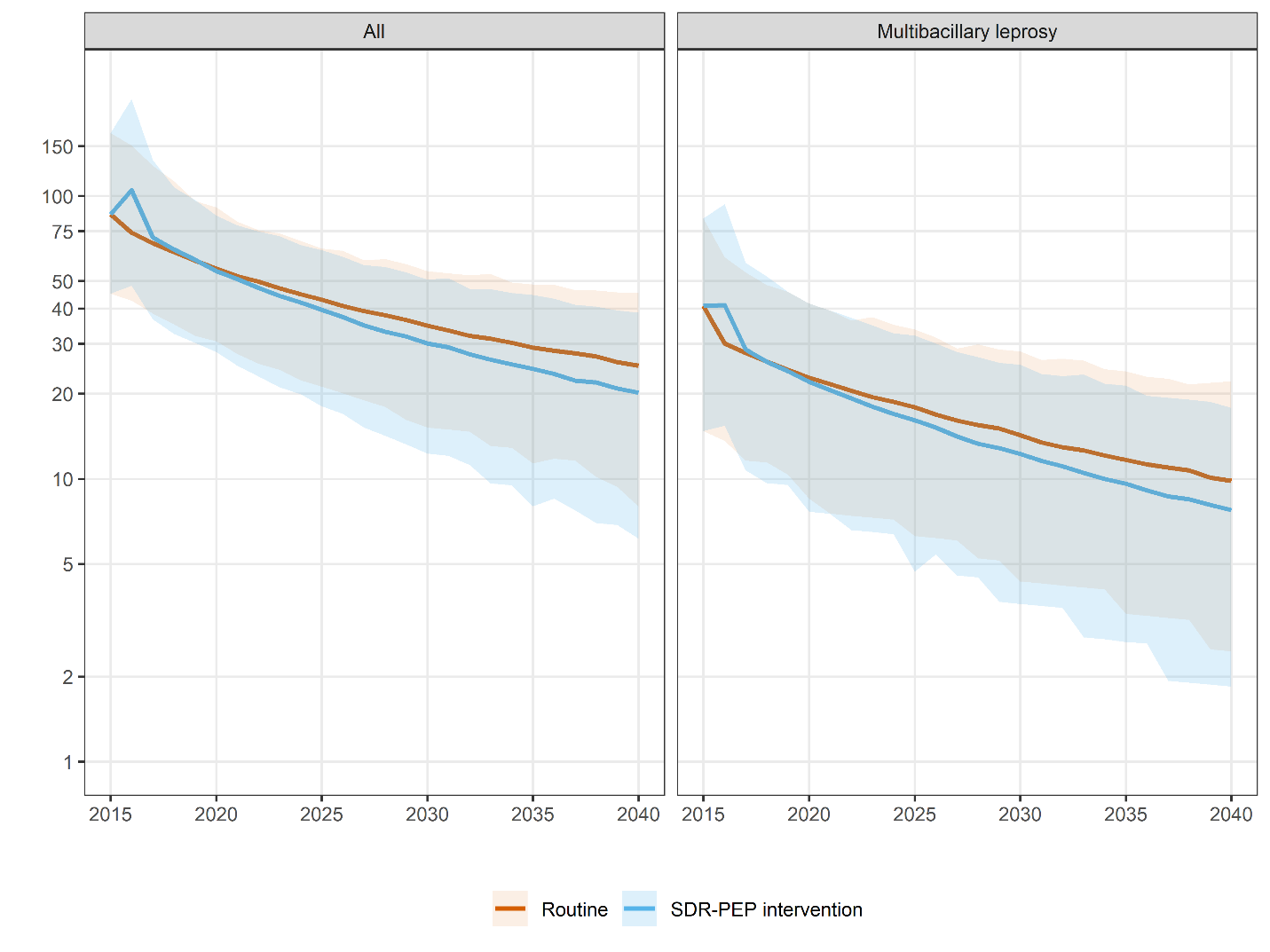


**S3 Fig. Predicted impact of SDR-PEP intervention in Dadra Nagar & Haveli India**

Predicted trends of a continuation of the routine programme and the SDR-PEP intervention. Results are the average of 1000 runs. The shaded area is the 95% uncertainty interval, representing the uncertainty in parameter estimates.
