# Supplementary material for: Leprosy post-exposure prophylaxis in the Indian health system: A cost-effectiveness analysis
Source: PLoS Negl Trop Dis. 2020 Aug 4;14(8):e0008521. doi: 10.1371/journal.pntd.0008521 (PMC7428216; doi:10.1371/journal.pntd.0008521)
Supplement: S4 Fig — (DOCX) [file pntd.0008521.s004.docx]

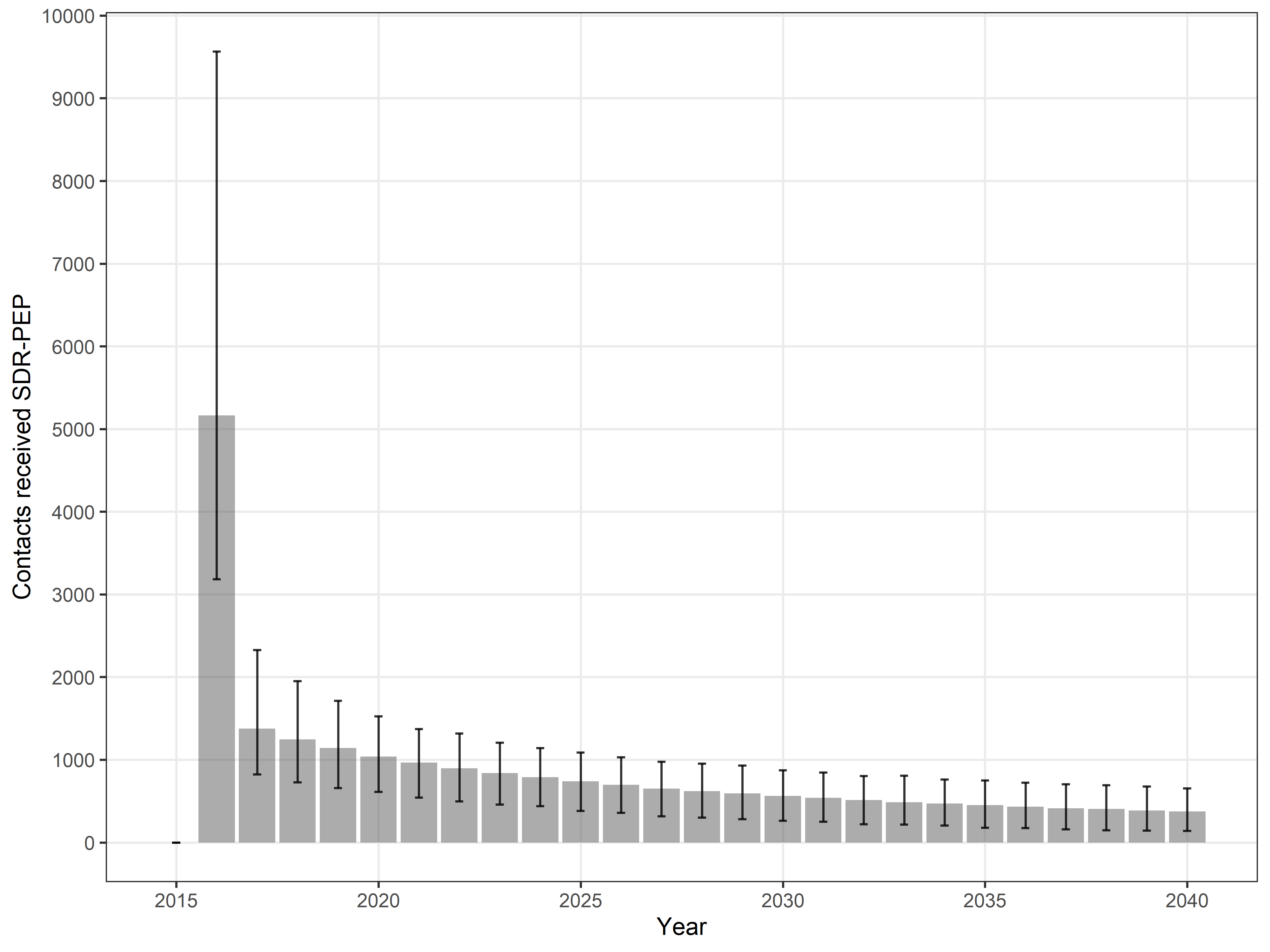


**S4 Fig. Contacts received SDR-PEP**

Model outcomes are represented by means (bars) and 95% uncertainty intervals (error bars). Predictions were made for the years 2015 to 2040.
