# Supplementary material for: Leprosy post-exposure prophylaxis in the Indian health system: A cost-effectiveness analysis
Source: PLoS Negl Trop Dis. 2020 Aug 4;14(8):e0008521. doi: 10.1371/journal.pntd.0008521 (PMC7428216; doi:10.1371/journal.pntd.0008521)
Supplement: S1 Table — (DOCX) [file pntd.0008521.s005.docx]

**S1 Table.** Pre and post LPEP comparison of Dadra and Nagar Haveli (DNH) on demography, socioeconomics and epidemiology.

| **DNH Indicators** | **2011** | |
| --- | --- | --- |
| *Demographic and socio-economic indicators* | | |
| Population | 343,709 | |
| Population growth (1991-2011) | 57.4 % | |
| Females (per 1000 males) | 774 | |
| Scheduled tribes† | 51.9% | |
|  | **Pre-LPEP (2014-15)** | **Post LPEP (2018-19)** |
| *Epidemiology* | | |
| Population* | 409,015 | 424,394 |
| New leprosy cases detected | 318 | 75 |
| NCDR (per 100 000 per year) | 77.7 | 59.9 |
| Grade II disability in new cases | 2% (4.89 per million) | Not available |
| New child cases (age <14 years) | 66 (20.7%) | 16 (Not available %) |
| New female cases | 57.5% | Not available |
| Prevalence/Registered patient rate (per 10,000 per year) | 5.0 | 3.55 |
| Multibacillary (MB) leprosy | 35.8% | Not available |

Sources: Census 2011 and NLEP reports

NCDR: new case detection rate

† The Scheduled Castes (SCs) and Scheduled Tribes (STs) are officially designated groups of historically disadvantaged indigenous people in India.
