# Supplementary material for: Leprosy post-exposure prophylaxis in the Indian health system: A cost-effectiveness analysis
Source: PLoS Negl Trop Dis. 2020 Aug 4;14(8):e0008521. doi: 10.1371/journal.pntd.0008521 (PMC7428216; doi:10.1371/journal.pntd.0008521)
Supplement: S2 Table — (DOCX) [file pntd.0008521.s006.docx]

**S2 Table**. **Epidemiologic data and parameters to quantify the model**

| **Data** | **Years** | **Source** |
| --- | --- | --- |
| **Epidemiologic data** |  |  |
| New case detection rate | 1995–2015 | NLEP India [1] |
|  |  |  |
| MB / PB ratio of new cases | 1995-2015 | NLEP India [1] |
| BCG coverage | 1980-2011 | WHO [2] |
| **Parameters** | **Value** | **Source** |
| **Natural history of infection** |  |  |
| Proportion susceptible | 20% | Assumption |
| MB / PB ratio | 26 / 76 | NLEP India [1] |
| PB subclinical duration mean | 4.2 years; SD =1.9 (gamma distributed) | Fischer et al. 2010 & Fine 1982 [3, 4] |
| PB self-healing rate | 20% per year | Fischer et al. 2010 & Sirumban et al. 1988 [3, 5] |
| MB subclinical duration mean | 11.1 years; SD = 5.0 (gamma distributed) | Fischer et al. 2010 & Fine 1982 [3, 4] |
| **Treatment** |  |  |
| MDT use | 1990 onwards |  |
| MDT relapse rate | - 1. per year   To MB: 90%  To PB: 10% |  |
| **Transmission** |  |  |
| Infectivity function  PB  Asymptomatic MB  Symptomatic MB | 0  Linear from 0 to 1  1 | Meima et al. 2004 [6] |
| Transmission rate  General population  Within households | (3, 7)^a^  0.98 | Calibrated ^a^  Fischer et al. 2010 [3] |
| **Control** |  |  |
| Passive case detection delays  Years of improved detection delay  *Detection delay function:*  $DD\left( t \right)=\left( \frac{max-min}{1+e^{b\cdot(t-mid)}} \right)+min$ | 1995, 1998, 1999, 2001, 2011, 2012 | Based on data from NLEP India [1] |
| Min  Max  Mid  b (slope) | 2  (20, 40)^a^  (0.05, 2)^a^  (0, 9)^a^ | Calibrated ^a^ |
| Survey  Year  Coverage | 2002  (0.05, 0.2) | Based on data from NLEP India [1]  Calibrated ^a^ |
| BCG protection | 60% | Schuring et al. 2009 [7] |
| ^a^ Calibrated to match modelled leprosy new case detection rate trend to data. We randomly drew parameter values from uniform distributions within these intervals. The model was run with these parameter values, which were accepted if the fit was good. The goodness of fit was assessed using a log-likelihood assuming a Poisson distribution. We repeated this until we had 1,000 parameter combinations that produced a good fit. Uncertainty intervals, which reflect uncertainty in the parameter values, were calculated by discarding the 2.5% highest and lowest values. | | |
|  | | |

1. Programme), N.N.L.E., *NLEP - Progress report* 2017, Central Leprosy Division: New Delhi.

2. WHO. *Reported estimates of BCG coverage*. [cited 2015; Available from: <http://apps.who.int/immunization_monitoring/globalsummary/timeseries/tscoveragebcg.html>.

3. Fischer, E., et al., *Different mechanisms for heterogeneity in leprosy susceptibility can explain disease clustering within households.* PLoS One, 2010. **5**(11): p. e14061.

4. Fine, P.E., *Leprosy: the epidemiology of a slow bacterium.* Epidemiological Review, 1982. **4**: p. 161-188.

5. Sirumban, P., A. Kumar, and P.N. Neelan, *Healing time in untreated paucibacillary leprosy: a cross-sectional study.* Int J Lepr Other Mycobact Dis, 1988. **56**(2): p. 223-7.

6. Meima, A., et al., *The future incidence of leprosy: a scenario analysis.* Bull World Health Organ, 2004. **82**(5): p. 373-80.

7. Schuring, R.P., et al., *Protective effect of the combination BCG vaccination and rifampicin prophylaxis in leprosy prevention.* Vaccine, 2009. **27**(50): p. 7125-8.
