# Supplementary material for: Leprosy post-exposure prophylaxis in the Indian health system: A cost-effectiveness analysis
Source: PLoS Negl Trop Dis. 2020 Aug 4;14(8):e0008521. doi: 10.1371/journal.pntd.0008521 (PMC7428216; doi:10.1371/journal.pntd.0008521)
Supplement: S3 Table — (DOCX) [file pntd.0008521.s007.docx]

**S3 Table**. **Demographic data and parameters to quantify the model**

| **Demographic data** |  |  |
| --- | --- | --- |
| **Data** | **Years** | **Source** |
| Population growth | 1901–2011 | Census India [1] |
|  |  |  |
| Fraction married | 1991, 2001, 2011 | Census India [2] |
| Survival rates | 1995, 1999, 2003, 2007, 2011 | Census India [3] |
| Fertility rates | 1990, 1993, 1996, 1999, 2006, 2011 | Census India [3] |
| Age distribution | 2011 | Census India [2] |
| Distribution of household size | 2011 | Census India [2] |
| **Household movement parameters** | | |
| **Parameter** | **Value** | **Source** |
| Fraction random movement | 0.71 | Calibrated ^a^ |
| Fraction creates own household | 0 | Assumption |
| Household size to move to | Start =0, End = 4, Max =3 (Triangular distributed) | Calibrated ^a^ |
| Fraction of married couple creating own household | 0.25 | Fischer et al. 2010 [4] |
| Time until splitting of a married household from parental household | Mean = 12 (Exponentially distributed) | Fischer et al. 2010 [4] |
| Fraction single widow(er)s moving back to children | 1.0 | Fischer et al. 2010 [4] |
| ^a^ Calibrated to match modelled household size distribution to data | | |
|  | | |

1. Census India. *Variation in Population since 1901*. [cited 2018 July 11]; Available from: <http://censusindia.gov.in/Census_Data_2001/India_at_glance/variation.aspx>.

2. Census India. *Tabulations Plan of Census Year - 2011*. [cited 2018 July 11]; Available from: <http://www.censusindia.gov.in/DigitalLibrary/TablesSeries2001.aspx>.

3. Census India. *Sample Registration System*. [cited 2018 July 11]; Available from: <http://www.censusindia.gov.in/2011-Common/Sample_Registration_System.html>.

4. Fischer, E., et al., *Different mechanisms for heterogeneity in leprosy susceptibility can explain disease clustering within households.* PLoS One, 2010. **5**(11): p. e14061.
